# Clinical predictive value of manual muscle strength testing during critical illness: an observational cohort study

**DOI:** 10.1186/cc13052

**Published:** 2013-10-10

**Authors:** Bronwen A Connolly, Gareth D Jones, Alexandra A Curtis, Patrick B Murphy, Abdel Douiri, Nicholas S Hopkinson, Michael I Polkey, John Moxham, Nicholas Hart

**Affiliations:** 1 Department of Asthma, Allergy & Respiratory Science, Division of Asthma, Allergy and Lung Biology, King’s College London, Great Maze Pond, London SE1 9RT, UK; 2 Guy’s & St Thomas’ NHS Foundation Trust and King’s College London, National Institute of Health Research Biomedical Research Centre, Great Maze Pond, London SE1 9RT, UK; 3 Lane Fox Clinical Respiratory Physiology Research Unit, St Thomas’ Hospital, Guy’s & St Thomas’ NHS Foundation Trust, Westminster Bridge Road, London SE1 7EH, UK; 4 Physiotherapy Department, St Thomas’ Hospital, Guy’s & St Thomas’ NHS Foundation Trust, Westminster Bridge Road, London SE1 7EH, UK; 5 Department of Public Health Sciences, King’s College London, 42 Weston Street, London SE1 3QD, UK; 6 National Heart and Lung Institute, National Institute of Health Research Respiratory Biomedical Research Unit, Royal Brompton and Harefield NHS Foundation Trust & Imperial College London, Sydney Street, London SW3 6NP, UK

## Abstract

**Introduction:**

Impaired skeletal muscle function has important clinical outcome implications for survivors of critical illness. Previous studies employing volitional manual muscle testing for diagnosing intensive care unit-acquired weakness (ICU-AW) during the early stages of critical illness have only provided limited data on outcome. This study aimed to determine inter-observer agreement and clinical predictive value of the Medical Research Council sum score (MRC-SS) test in critically ill patients.

**Methods:**

*Study 1:* Inter-observer agreement for ICU-AW between two clinicians in critically ill patients within ICU (*n* = 20) was compared with simulated presentations (*n* = 20). *Study 2:* MRC-SS at awakening in an unselected sequential ICU cohort was used to determine the clinical predictive value (*n* = 94) for outcomes of ICU and hospital mortality and length of stay.

**Results:**

Although the intra-class correlation coefficient (ICC) for MRC-SS in the ICU was 0.94 (95% CI 0.85–0.98), κ statistic for diagnosis of ICU-AW (MRC-SS <48/60) was only 0.60 (95% CI 0.25–0.95). Agreement for simulated weakness presentations was almost complete (ICC 1.0 (95% CI 0.99–1.0), with a κ statistic of 1.0 (95% CI 1.0–1.0)). There was no association observed between ability to perform the MRC-SS and clinical outcome and no association between ICU-AW and mortality. Although ICU-AW demonstrated limited positive predictive value for ICU (54.2%; 95% CI 39.2–68.6) and hospital (66.7%; 95% CI 51.6–79.6) length of stay, the negative predictive value for ICU length of stay was clinically acceptable (88.2%; 95% CI 63.6–98.5).

**Conclusions:**

These data highlight the limited clinical applicability of volitional muscle strength testing in critically ill patients. Alternative non-volitional strategies are required for assessment and monitoring of muscle function in the early stages of critical illness.

## Introduction

Skeletal muscle weakness is a common complication of critical illness and a major factor influencing both short-term and long-term clinical outcome [1-5]. This has driven, as a priority, the development of the clinical concept of ICU-acquired weakness (ICU-AW). ICU-AW has a reported prevalence of up to 65% [6], with observational studies showing associations with prolonged weaning, delayed rehabilitation, increased hospital length of stay (LOS) and increased mortality [6-13]. However, such observational cohort studies do not necessarily demonstrate a causal relationship.

Diagnostic criteria for ICU-AW are based on clinical examination [14]. Whilst further subclassification of critical illness neuromyopathy can be achieved using detailed nonvolitional electrophysiological investigations, this can be technically challenging in the ICU because it requires skilled personnel for both assessment and interpretation [15]. Simple tests with potentially greater clinical applicability have been proposed. A measure of global peripheral muscle strength, the Medical Research Council sum score (MRC-SS), which ranges from 0 (complete paralysis) to 60 (normal strength) [16], has been widely used, with scores less than 48 providing the basis for diagnosing ICU-AW [14]. As with all volitional measures of muscle strength, however, a patient’s inability to perform the test or a low score may occur as a result of nonmuscular factors, such as impaired cognition, reduced consciousness level and poor motivation. Furthermore, the ordinal, nonlinear nature of grading muscle strength results in potential variability between clinicians in both application of testing and interpretation of results [17]. These caveats have led to contrasting data for diagnosing ICU-AW within the ICU, as well as to variability in interobserver agreement of the MRC-SS in ICU patients with differing levels of weakness [18,19].

There are no published studies to date that have reported the clinical applicability of the MRC-SS in a general ICU population, in particular the clinical usefulness of the MRC-SS in predicting ICU and in-hospital patient outcomes. In the current study, we investigated (1) interobserver agreement regarding ICU-AW in critically ill patients in the ICU and regarding simulated weakness, (2) the clinical predictive value of ability to perform the MRC-SS test at awakening and (3) the clinical predictive value of an MRC-SS less than 48, which is considered diagnostic of ICU-AW [20].

## Materials and methods

### Study design and ethical approval

We conducted a two-part, observational, single-centre study in a 30-bed mixed medical and surgical ICU in a university teaching hospital. In study 1, we determined interobserver agreement regarding MRC-SS in ICU patients and simulated weakness presentations. Local ethical review board approval was granted (London–Westminster Research Ethics Committee 09/H0802/80). Written informed consent was obtained from all participants. In study 2, we investigated the clinical predictive value of ability to perform MRC-SS at awakening and the degree to which MRC-SS is indicative of ICU-AW. The local hospital ICU audit committee considered study 2 an evaluation of clinical service for which specific ethical approval was not required.

### Patients

Patients 18 years of age and older who had been invasively ventilated for 48 or more hours were eligible for inclusion. Exclusion criteria included neurological weakness, requirement for acute noninvasive ventilation, pregnancy, malignancy, palliation-only orders and those admitted for routine overnight postoperative surgical recovery. Separate patient cohorts were recruited for studies 1 and 2.

### Screening for awakening and assessment of peripheral muscle strength

For studies 1 and 2, the consciousness level of patients was determined using the Richmond Agitation Sedation Scale [21], with a score from −1 to +1 being indicative of wakefulness. Awake patients were then required to demonstrate a positive response to simple one-stage commands [6,7,10]. Successful completion of commands was followed by muscle strength assessment using the MRC-SS—a six-point grading scale ranging from 0 (no visible contraction) to 5 (normal power) applied to six upper- and lower-limb muscle groups bilaterally [16] (Additional file 1: S2, Table S2a). ICU-AW was defined as an MRC-SS less than 48 out of a possible score of 60 [7-9,13,22].

### Clinical examiners

Clinical examiners for the MRC-SS were two specialist physiotherapists (GJ and AC) with extensive clinical expertise in rehabilitation of critically ill patients, including muscle strength assessment using the MRC-SS. A standardised protocol for performing the MRC-SS was followed at all times during testing (Additional file 1: S2, Tables S2b and S2c). Given the volitional nature of manual muscle testing, strong verbal encouragement was provided during all strength assessments. Each patient was tested in the same position by the examiners.

### Study 1: Investigation of interobserver agreement in ICU patients and simulated weakness

A pragmatic sample size of 20 patients was chosen for this observational study. Sequential eligible, consenting patients were recruited depending on the working schedules and availability of both examiners over a three-month period. MRC-SS testing was performed by both examiners individually, separated by 30 minutes. Initial testing order between examiners of the first patient was randomly assigned by concealed envelope, and subsequent patient testing orders followed an alternating pattern. The MRC-SS value obtained by the first testing clinician on each occasion was defined as the ‘reference’ score for the simulated presentation. One healthy volunteer, trained comprehensively in the MRC-SS, simulated these 20 reference scores in a random order, and the reference scores were rescored by both clinicians (Additional file 1: S3). Clinician order of testing was randomised for the first presentation, and an alternating pattern was followed thereafter. At each stage, the clinicians were blinded to each other’s scoring and to the reference score.

### Study 2: Investigation of the clinical predictive value of Medical Research Council sum score

Daily screening of ICU patients for eligibility and suitability for MRC-SS testing occurred over a three-month period. MRC-SS at awakening, defined as the first occasion when an MRC-SS could be measured, and at seven days postawakening, were compared against outcomes of ICU and hospital mortality and LOS. Awakening scores were used to determine association with prospective outcomes. Prolonged LOS was defined *a priori* as longer than 14 days for ICU LOS and longer than 28 days for in-hospital LOS.

### Statistical analysis

In study 1, interobserver agreement between clinicians for the MRC-SS in ICU patients and simulated presentations was determined using intraclass correlation coefficients (ICCs), which were calculated using two-way random effects for absolute agreement [23], and percentage agreement for total MRC-SS (total number of exact MRC-SS measurements divided by total number). Level of agreement for the binary outcome of ICU-AW (MRC-SS <48;≥48) was determined using Cohen’s κ statistic with a grading system from ‘poor’ to ‘complete’ agreement [24]. Additional details of the analysis of interobserver agreement are provided in Additional file 1: S4. In study 2, Fisher’s exact test was used to determine associations between MRC-SS outcomes (ability to perform the test and scores less than 48 on a scale of 60) and clinical outcomes (ICU and hospital mortality and LOS). Subsequent analysis of test characteristics (sensitivity, specificity, positive predictive value (PPV) and negative predictive value (NPV)) was then performed with a cutoff of 75% used to define clinically acceptable results. We additionally performed receiver-operator curve (ROC) analysis on MRC-SS measurements at awakening for each clinical outcome to assess sensitivity and specificity at levels of MRC-SS from 0 to 60. Parametric data are presented as means ± SD, nonparametric data are presented as medians (IQR) and appropriate testing was applied. A *P* value less than 0.05 was considered statistically significant. Data analyses were performed using SPSS Statistics software (SPSS, Inc, Chicago, IL, USA), GraphPad Prism version 5 for Windows software (GraphPad Software, La Jolla, CA USA) and Confidence Interval Analysis (CIA) for Windows software (University of Southampton, UK).

## Results

### Interobserver agreement regarding Medical Research Council sum scores of ICU patients

**Figure 1:**
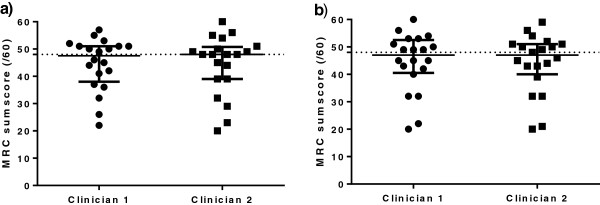
**Medical Research Council sum score for clinician testing of critically ill patients and simulated presentations. a)** Medical Research Council sum scores (MRC-SSs) in critically ill patients from each clinician. **b)** MRC-SSs in simulated presentations from each clinician. Error bars indicate medians and IQRs. Dotted lines indicate cutoff value of 48 on a 60-point scale to indicate diagnosis of ICU-acquired weakness. Abbreviations: MRC-SS = Medical Research Council sum score.

The demographic and clinical data from the cohort (*N* = 20) are shown in Table 1. The median (IQR) ICU LOS prior to MRC-SS testing was 24.0 days (6.8 to 43.3). At the time of testing, 45% of patients were receiving invasive mechanical ventilation (MV). All muscle groups were tested on all occasions. The median MRC-SSs for each testing clinician were 48 (IQR: 39 to 51; range: 22 to 57) and 48 (IQR: 38 to 51; range: 22 to 60) (Figure 1). Table 2 reports the MRC-SSs obtained during testing by both clinicians. Median time between testing by clinicians was 30 minutes (IQR: 29 to 33). The maximum difference in MRC-SS measurements for any one patient was 7, and the agreement between clinicians’ scores was 15.0%. The ICC was 0.94 (95% confidence interval (CI): 0.85 to 0.98), and the κ statistic for agreement on the diagnosis of ICU-AW was 0.60 (95% CI: 0.25 to 0.95). The results of interobserver agreement for individual muscle group scores, and the comparison between clinicians for the ICU cohort, can be found in Additional file 1: S5, Tables S5a and S5b.

**Table 1:** **Demographic, admission and clinical data from study 1 and study 2**^**a**^

	**Range**	
**Characteristics**	**Study 1: MRC-SS interobserver agreement (*n *** **= 20)**	**Study 2: MRC-SS clinical predictive value (*n *** **= 94)**
Age (years)	67.5 (51.8 to 75.0)	66.0 (54.8 to 76.3)
Gender (M:F), *n*	12:8	64:30
APACHE II score	19.5 (15.5 to 24.0)	17.0 (15.0 to 22.0)
Admission type		
Medical (%)	70.0	78.7
Surgical (%)	30.0	21.3
Comorbidities		
Chronic respiratory disease (%)	50.0	27.7
Cardiac disease (%)	65.0	55.3
Chronic renal disease (%)	5.0	4.0
Chronic liver disease (%)	0.0	10.6
Total MV (days)	25.5 (21.0 to 44.0)	7.0 (4.0 to 16.0)
Total MV prior to MRC-SS testing (days)	21.0 (6.8 to 42.0)	5.0 (3.0 to 9.5)^b^
ICU LOS total (days)	33.5 (25.5 to 58.0)	11.0 (6.0 to 25.3)
ICU LOS prior to MRC-SS testing (days)	24.0 (6.8 to 43.3)	N/A
Hospital LOS total (days)	52.5 (31.5 to 85.3)	27.0 (11.8 to 50.0)
Hospital LOS prior to MRC-SS testing (days)	23.5 (7.5 to 43.8)	N/A

^a^APACHE II: Acute Physiological and Chronic Health Evaluation II, LOS: length of stay, MRC-SS: Medical Research Council sum score, MV: mechanical ventilation, N/A: not applicable. Data are expressed as medians (IQR) (*N* = 20). For comorbidities, values reflect percentage of the cohort with specific organ disease with overlap across categories. Hence, there are sums greater than 100%. ^b^*n* = 65 for number of patients with MRC-SS at awakening.

**Table 2:** Interobserver agreement regarding Medical Research Council sum scores (on a scale of 60) in 20 ICU patients and 20 simulated presentations

**Patient**	**ICU patients**		**Simulated weakness presentations**		
	**Clinician 1**	**Clinician 2**	**Reference score**	**Clinician 1**	**Clinician 2**
1	46	48	20	20	20
2	36	32	44	43	44
3	51	48	22	22	21
4	26	20	60	60	59
5	52	49	56	56	56
6	51	45	52	53	51
7	50	48	32	32	32
8	45	49	50	49	50
9	57	60	32	32	32
10	37	39	50	50	50
11	55	54	42	42	43
12	22	23	54	54	54
13	49	55	39	40	39
14	44	44	45	45	43
15	50	50	45	45	45
16	41	48	48	49	48
17	42	39	46	45	46
18	53	56	49	49	49
19	32	29	51	53	52
20	51	51	51	51	51

### Interobserver agreement for simulated Medical Research Council sum score presentations

The data were analysed in a manner similar to that used previously. MRC-SS measurements by the two clinicians were 47 (IQR: 40 to 51; range: 20 to 60) and 47 (41 to 53; range: 20 to 59) (Figure 1). Table 2 reports the MRC-SSs obtained during testing by both clinicians against the simulated reference score. Ten reference MRC-SSs were less than 48, including four that were less than 36. The maximum difference between clinicians’ MRC-SS measurements for any individual presentation was 2 with 45.0% agreement. The ICC for simulated MRC-SS values was 1.0 (95% CI: 0.99 to 1.0). Complete agreement for diagnosis of ICU-AW on the basis of simulated presentations was evident (κ statistic: 1.0 (95% CI: 1.0 to 1.0)). The results for interobserver agreement regarding individual muscle group scores, and for comparisons between clinicians and against the reference score, are reported in Additional file 1: S6, Tables S6a and S6b; and S7, Tables S7a and S7b.

### Predictive value of ability to perform Medical Research Council sum score testing at awakening

**Figure 2:**
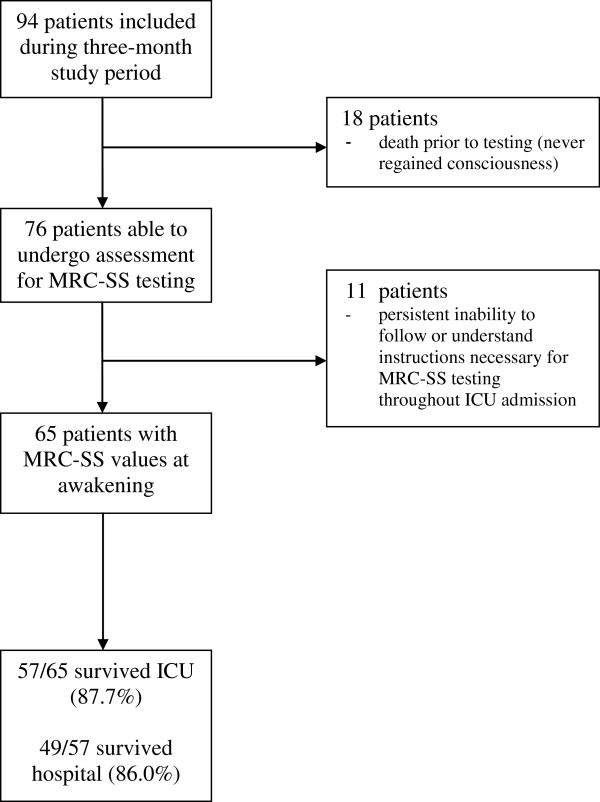
**Flow diagram of patient enrolment and evaluation throughout the study.** MRC-SS: Medical Research Council sum score.

Ninety-four patients were eligible for enrolment during the three-month study period (Figure 2). The baseline demographic data for the cohort are reported in Table 1*.* Eighteen patients died prior to any testing, and eleven patients were consistently unable to perform (UTP) MRC-SS testing throughout their ICU stay because of cognitive impairment. Sixty-five patients were able to undergo MRC-SS at awakening. When the cohort was categorised into able to perform (ATP) and UTP MRC-SS testing at awakening patient groups, significant differences between groups were evident across the parameters of age (ATP 35.3 ± 14.9 years vs. UTP 60.6 ± 20.0 years; *P* < 0.0001), illness severity at time of ICU admission (Acute Physiological and Chronic Health Evaluation II score) (ATP 18.5 ± 5.1 vs. UTP 14.9 ± 4.6; *P* = 0.03) and hospital LOS (ATP 33 days (14.5 to 55.5) vs. UTP 15 days (7.0 to 37.0); *P* = 0.02). Groups were similar for gender, ICU LOS and total MV days. Duration of MV prior to awakening MRC-SS was five days (3 to 9.5) in the ATP group and 0.0 days (0.0 to 6.5) following awakening MRC-SS testing. In the UTP group, the number of attempted MRC-SS assessments was 4.0 (2.0 to 8.0). ICU mortality rates were 12.3% and 0.0% and in-hospital mortality rates were 24.6% and 18.2% for the ATP and UTP groups, respectively. We performed Fisher’s exact testing to examine any association between ability to perform the test at awakening and ICU and in-hospital mortality and LOS. The results of all tests were nonsignificant, and therefore further analysis of test characteristics was not considered appropriate.

At day 7, 45 of the 65 patients with awakening scores had been discharged from the ICU (8 patients had died, and 37 patients had been transferred to the ward or repatriated), and 6 were unable to perform the test. Fourteen patients had MRC-SSs of 33.5 (22.3 to 44.8). Eleven had ICU-AW (MRC-SSs less than 48). Owing to the small numbers of patients, further analysis of this cohort was not considered appropriate.

### Predictive value of a Medical Research Council sum scores less than 48 and 48 or higher at awakening

Of the 65 patients with MRC-SSs at awakening, 33 had scores of 0 to 36 (50.8%), 15 (23.1%) scored 37 to 47 and 17 (26.1%) scored 48 or higher. The prevalence of ICU-AW (MRC-SS less than 48) in the cohort was 73.9% (M:F ratio 35:13). There was no association between MRC-SS and ICU and hospital mortality (*P* = 0.67 and *P* = 0.53, respectively), and therefore further analysis of test characteristics was not performed. However, a significant association was found for ICU and hospital LOS (*P* = 0.004 and *P* = 0.04, respectively). The clinical predictive value of MRC-SS less than 48 at awakening was therefore determined (Table 3). Using a cutoff of 75%, high sensitivity was evident for ICU and hospital LOS. Specificity and PPV were poor across both parameters, with a high NPV evident for ICU LOS.

**Table 3:** **Clinical predictive value of Medical Research Council sum scores less than 48 at awakening**^**a**^

	**ICU LOS (≤14 days and >14 days)**		**Hospital LOS (≤28 days and >28 days)**	
**Measurement**	**%**	**95% CI**	**%**	**95% CI**
Sensitivity	92.9	76.5–99.1	84.2	68.7–94.0
Specificity	40.5	24.8–57.9	40.7	22.4–61.2
PPV	54.2	39.2–68.6	66.7	51.6–79.6
NPV	88.2	63.6–98.5	64.7	38.3–85.8

^a^CI: confidence interval, LOS: length of stay, NPV: negative predictive value, PPV: positive predictive value.

ROC analysis was performed on the 65 awakening MRC-SS measurements for each clinical outcome to assess sensitivity and specificity at levels of MRC-SS from zero to 60. Further data from this analysis can be found in Additional file 1: S8, Table S8. The greatest sensitivity was observed at an MRC-SS less than 35 (64.3%) with 64.9% specificity (area under the curve (AUC): 0.69 (95% CI: 0.56 to 0.82)) for ICU LOS, and the greatest specificity was observed at an MRC-SS less than 29.5 (70.2%) with 62.5% sensitivity (AUC: 0.63 (95% CI: 0.42 to 0.83)) for ICU mortality, albeit that these ‘cutoffs’ have limited clinical usefulness.

### Relationship between Medical Research Council sum score at awakening and handgrip strength and physical function

Data detailing the relationship between MRC-SS at awakening and handgrip strength and physical function at ICU discharge are reported in Additional file 1: S1, Table S1. Patients diagnosed with ICU-AW demonstrated reduced handgrip strength compared to those without ICU-AW. However, only a weak direct relationship between MRC-SS at awakening and handgrip strength at ICU discharge was demonstrated. Furthermore, only a weak correlation was shown between MRC-SS and two common measures of physical function, with no difference in physical function observed between groups with or without a diagnosis of ICU-AW.

## Discussion

We have shown that, despite high interobserver agreement regarding MRC-SSs between two expert clinicians who assessed ICU patients and between their evaluations and simulated presentations of weakness, there was only moderate agreement for the diagnosis of ICU-AW in the ICU cohort. This confirms that interobserver agreement for the diagnosis of ICU-AW is a consequence of patient rather than clinician variability during testing, which wholly limits the clinical value of the test. In addition, almost one-third of ICU patients were unable to perform the MRC-SS test, but there were no relationships observed between the ability to perform the MRC-SS at awakening and mortality and LOS. Furthermore, an MRC-SS less than 48, indicative of ICU-AW, had limited PPV and NPV for a hospital LOS of more than four weeks, albeit that a high NPV was observed for an ICU LOS of more than two weeks. Clinically, this suggests that an MRC-SS less than 48 had poor predictive value but that an MRC-SS greater than 48 predicted a more favourable outcome. These data highlight the limitations and clinical usefulness of the MRC-SS as a marker of ICU-AW in a general ICU population in the early stages of critical illness.

### Critique of the method

Determining the ideal protocol for establishing interobserver agreement regarding the MRC-SS in critically ill patients within the ICU and controlling for potentially confounding variables is challenging. We separated patient testing by 30 minutes to minimise the effect of clinical fluctuation and avoid patient exhaustion, albeit that a longer duration may have been required for this purpose. Specifically, we elected not to collect measurements at any intervening time points, as unpredictable fluctuations in the clinical status of patients remains a constant limiting factor in the reliability of MRC-SSs. Furthermore, we adopted a standardised protocol for MRC-SS measurement according to patient position to limit clinician variability, which has not previously been reported. Despite these approaches, we acknowledge that patient-related factors, in particular pain, may have influenced the clinician’s ability to perform the assessment, regardless of the patient’s successfully meeting screening criteria for alertness and cognitive ability on each occasion. A small cohort of patients were unable to complete MRC-SS testing because of persistent inability to understand or follow the necessary instructions, suggesting that screening using simple one-stage commands may be inadequately sensitive to detect cognitive ability sufficient for MRC-SS assessment. More thorough assessment of delirium and complex cognitive ability may have addressed this problem [25], but we aimed to reflect the common approach employed in previous studies [6,7,10,19]. We also acknowledge that we did not document sedation dose and opiate requirements, but the absence of this information should not detract from the fact that the inability to perform the test lacked clinical utility in predicting outcome and follows the methodology of previous studies in this area [4,7,10]. Indeed, there were no patients within the cohort whose causal ICU admission diagnosis physically precluded them from completing testing, for example, secondary to trauma. Outcomes of mortality and LOS were selected based on findings from previous observational cohort studies in which researchers investigated ICU-AW, diagnosed on the basis of the MRC-SS, and clinical course [6,7,10]. However, we recognise that these outcomes are influenced by multiple factors in critically ill patients and that peripheral muscle strength may not represent the most relevant diagnostic tool. We acknowledge that these data need to be interpreted carefully, as only one-fourth of patients with awakening MRC-SS values did not have ICU-AW.

In the current study, *awakening* was defined as the first occasion on which MRC-SS could be obtained from a patient. In contrast to the original study of De Jonghe *et al.*[10], who defined ICU-AW as an MRC-SS less than 48 at seven days postawakening, we found that, owing to high rates of patient discharge from the ICU by this time, scores at day 7 were considerably less useful. Specifically, the majority of patients in the ICU at day 7 postawakening demonstrated ICU-AW, but these patients comprised a small subgroup of the general ICU patient cohort studied (15%), and thus analysis of these data was extremely limited. This reflects the change in clinical ICU practice toward earlier discharge as a result of implementation of structured weaning and reduced-sedation protocols, as well as a growing culture of early mobilisation.

### Interobserver agreement

Although interobserver agreement was determined in a relatively small sample of ICU patients recovering from critical illness in our present study, thus limiting the application of these findings to the wider ICU population, our approach allowed testing in a relatively stable group of patients with potentially less clinical fluctuation whilst they were still in the ICU. However, only moderate agreement regarding MRC-SSs less than 48, diagnostic of ICU-AW, was evident. The current subgroup shared clinical characteristics similar to those of a recently published data set that also demonstrated moderate levels of interobserver agreement regarding ICU-AW diagnosis [18]. Levels of agreement between clinicians were completely matched for MRC-SS and diagnosis of ICU-AW in simulated weakness presentations. Interobserver variability was therefore the result of patient-related variation in ability to perform the volitional MRC-SS rather than variability between clinicians in conducting the assessment. Whilst previously assumed, these results confirm the source of error in determining interobserver agreement of MRC-SS measurement to be patient variability and represent an important and novel aspect of the current research. We focussed on interobserver agreement of the MRC-SS, given that, in routine clinical practice, it is likely that more than one therapist is involved in the management of critically ill patients and that any potentially diagnostic measure requires consistency between clinicians. We therefore attempted to reduce interobserver bias by using experienced raters, but we acknowledge that, in clinical practice, greater variability in scoring may occur when carried out by clinicians with less experience.

### Clinical interpretation of Medical Research Council sum score

Although previous data have associated ICU-AW with poor clinical outcome [6-8,10], determining the test characteristics of the MRC-SS as an assessment tool has never previously been reported in the literature. The clinical interpretation of these data is important to clarify.

Inability to perform the test did not predict a poor outcome in terms of ICU and in-hospital mortality and LOS. Likewise, there was no relationship observed between preserved peripheral muscle strength (MRC-SS 48 or higher) and ICU-AW (MRC-SS less than 48) and mortality. Despite demonstration of an association between MRC-SS and ICU and hospital LOS, test characteristics revealed that, whilst higher scores predicted a favourable outcome, lower scores did not predict a poor clinical outcome. These observations are in principle similar to those our own group and others have made when using volitional measurements of respiratory muscle strength whereby a high value supports confirmation of preserved muscle strength and a low value is not necessarily representative of muscle weakness, but rather is related to ability to perform the test effectively [26-29]. Further analysis using ROCs to define an MRC-SS cutoff for each of the important clinical outcomes of ICU and in-hospital mortality and LOS failed to identify clinically meaningful values of the MRC-SS with satisfactory sensitivity and specificity. These data highlight the limitations in the robustness of the MRC-SS for use in day-to-day clinical practice for predicting outcome, albeit that the sample size in this study was probably too small to be definitive. These data support the development of alternative outcome measures for monitoring the progression of muscle-wasting and weakness in critically ill patients, which need to be correlated with physical performance. Recent data have demonstrated a reduction in quadriceps rectus femoris cross-sectional area during early critical illness measured using ultrasound [30], with muscle layer thickness being negatively correlated with LOS [31]. These simple nonvolitional and effort-independent tests have the potential for further clinical application in the ICU to provide physiologically more accurate and robust data regarding muscle structure and function during critical illness. It is rational to consider that physical function has a relationship with muscle-wasting, although this connection has yet to be proven in the post–critical care population. Such data would provide strong support for targeted exercise therapy and rehabilitation for those patents with significant muscle-wasting with the expectation of enhancing physical function.

### Comparison with previous studies

The moderate interobserver agreement regarding the diagnosis of ICU-AW and low PPV of ICU-AW in the current study was not unexpected. Inherent clinical variation and unpredictability during early critical illness highlight the major limitations of employing volitional testing in this population and affect reliability. Although original reports of MRC-SS testing by Kleyweg *et al.*[16] demonstrated high levels of interobserver reliability of the MRC-SS, this finding was in a cohort of recovering, stable patients with Guillain-Barré syndrome, albeit that the cohort included bedbound patients still requiring invasive ventilatory support. κ agreement levels of 88% reported by Fan *et al.*[32] and 68% by Hermans *et al.*[18] for stable recovery patients differ from those of 38% reported by Hough *et al.*[19] and 60% in our current study for the diagnosis of ICU-AW in patients assessed whilst in the ICU. Furthermore, similar to Hough *et al.*[19], we have demonstrated in the present that a significant proportion of patients were unable to perform MRC-SS testing. The current data challenge the clinical usefulness of the MRC-SS in ICU patients early in the course of critical illness.

## Conclusion

Clinicians should understand the limitations of using the MRC-SS to diagnose ICU-AW during the early stages of critical illness. Even when MRC-SS testing is performed by expert clinicians, the fluctuating clinical status of patients can significantly reduce test reliability. Furthermore, patient inability to perform the test and a score indicative of ICU-AW demonstrated limited clinical usefulness in considering outcome. The findings of the current study reflect the limitations of volitional strength testing, and thus alternative nonvolitional techniques are required to objectively assess and monitor patients.

## Key messages

• Volitional manual muscle strength testing has limited clinical applicability in critically ill patients.

• There was high interobserver agreement between two expert clinicians regarding MRC-SSs used to assess ICU patients, as well as with regard to simulated weakness presentations, but only moderate agreement regarding the diagnosis of ICU-AW.

• There was no relationship between MRC-SS and mortality.

• MRC-SSs less than 48, diagnostic of ICU-AW, have limited clinical value for predicting LOS.

• Nonvolitional techniques are required for the assessment and monitoring of muscle-wasting in the early stages of critically illness.

## Abbreviations

ATP: Able to perform; AUC: Area under the curve; ICC: Intraclass correlation coefficient; ICU-AW: ICU-acquired weakness; IQR: Interquartile range; LOS: Length of stay; MRC-SS: Medical Research Council sum score; NPV: Negative predictive value; PPV: Positive predictive value; ROC: Receiver-operator curve; UTP: Unable to perform.

## Competing interests

The authors declare that they have no conflicts of interest.

## Authors’ contributions

BC contributed to the design of the study, was responsible for data analysis and interpretation, and drafted, revised and agreed on the final manuscript version for submission. GJ and AC contributed to the study design, were responsible for data acquisition and contributed to manuscript revision. PM, NSH, MIP and JM contributed to data interpretation and manuscript revision. AD assisted with statistical analysis. NH contributed to the study design and data interpretation, was responsible for manuscript revision and agreed on the final version for submission. NH acts as the guarantor for the intellectual integrity of the work. All authors read and approved the final manuscript.

## Supplementary Material

Additional file 1 Supplemental digital content. Click here for file
