# Supplementary material for: Clinical predictive value of manual muscle strength testing during critical illness: an observational cohort study
Source: Crit Care. 2013 Oct 10;17(5):R229. doi: 10.1186/cc13052 (PMC4057053; doi:10.1186/cc13052)
Supplement: Additional file 1 — Supplemental digital content. [file cc13052-S1.docx]

**SUPPLEMENTAL DIGITAL CONTENT**

**Clinical Predictive Value of Peripheral Muscle Strength Testing**

**During Early Critical Illness: an Observational Cohort Study**

**S1. Relationship between Medical Research Council-Sum-score, physical function and handgrip strength**

Previous data has reported the relationship between Medical Research Council Sum-score (MRC-SS) as a measure of global peripheral muscle strength and handgrip strength ([1](#_ENREF_1), [2](#_ENREF_2)) but none with physical function. Such data would characterise levels of weakness on the MRC-SS in a clinical context, and define physical functional ability of patients diagnosed with ICU-AW (MRC-SS <48/60) and those without. We aimed to determine the relationship between MRC-SS at awakening and physical function and handgrip strength at ICU discharge.

Clinical examiners for measurements were two senior specialist physiotherapists (GJ, AC) with extensive clinical expertise in rehabilitation of critically ill patients including muscle strength assessment using the MRC-SS and handgrip dynamometry, and assessment of physical function. A standardised protocol for performing the MRC-SS was followed at all times during testing (*S2*). MRC-SS in ICU patients at awakening were analysed with results of physical function measures and handgrip strength,using dynamometry, performed within 72hours of ICU discharge to the ward. This time point was pragmatic to ensure availability of either observer to perform testing, and also to confirm clinical stability of patients once discharged from the ICU. Physical function measures included the Barthel scale ([3](#_ENREF_3)), a measure of functional activity performance commonly used in the in-patient setting covering aspects of personal care, mobility and transfers and the Elderly Mobility Scale (EMS) ([4](#_ENREF_4)), a validated tool for assessing mobility in frail, elderly subjects. It includes assessment of functional transfers, balance and gait performance that may be applicable to the early stages of the rehabilitation process for post ICU patients.

Significant positive, but moderate correlations were evident between MRC-SS and Barthel scale (r=0.4, p=0.005), EMS (r=0.4, p=0.005), and bilateral handgrip strength (left (r=0.5, p=0.0003), right (r=0.5, p<0.0001)). Physical function and handgrip strength were then compared according to ICU-AW diagnosis (MRC-SS <48/60; MRC-SS ≥48/60) (*Table S1*). Significant differences were evident between groups for left (p=0.04) and right (p=0.002) handgrip strength, but not for Barthel and EMS score.

**Table S1:**  Medical Research Council-Sum score at awakening and measures of handgrip strength and physical function at intensive care unit discharge

|  | **MRC-SS <48 (n=48)** | **MRC-SS ≥48 (n=17)** | **p** |
| --- | --- | --- | --- |
| **L HGD (kg)** | 7.0 (2.0-15.0) | 12.0 (9.0-24.0) | 0.02 |
| **R HGD (kg)** | 7 (2-13.5) | 16 (11-25) | 0.002 |
| **Barthel score (/20)** | 2 (0-8.5) | 8 (1-11) | 0.05 |
| **EMS (/20)** | 2 (0-8) | 6 (2-15) | 0.07 |

^Data are mean±SD or median (Interquartile range). p values derived from Mann-Whitney or unpaired t-test (*).^

^Barthel and EMS, higher scores indicate better physical function.^

^Abbreviations: MRC-SS = Medical Research Council-Sum score. EMS = Elderly Mobility Scale. L = left. R = right. HGD = handgrip dynamometry.^

Patients diagnosed with ICU-AW demonstrated reduced handgrip strength compared to those without ICU-AW, which is similar to previously reported data ([1](#_ENREF_1)). However, the current data only demonstrated a weak direct relationship between MRC-SS at awakening and handgrip strength at ICU discharge that is not wholly unexpected. This weak correlation is, in part, a consequence of both the different timings of assessment as well as the different muscle groups that are assessed during the different tests. MRC-SS testing does not measure distal muscle function such as hand muscle strength, which is the muscle group often affected early on as part of a motor neuropathy ([5](#_ENREF_5)). Furthermore, ability to perform handgrip dynamometry according to standard guidelines ([6](#_ENREF_6)) requires upper limb strength and thus upper limb weakness demonstrated by those patients with MRC-SS less than 48 would have been expected to influence the performance of this measure. We did not capture data in hand dominance and are unable to comment on the influence of this on our results.

Only a weak correlation was shown between MRC-SS and two common measures of physical function, which again is not unexpected. In addition to the different timings of the assessments, the MRC-SS is a composite score of peripheral muscle strength, based on single muscle group manoeuvres. It fails to capture the spectrum of complex motor tasks and interaction between skeletal muscle strength and endurance, balance, co-ordination and higher-level cognition required for complex physical function activities. Many activities assessed with the Barthel and EMS scales involve hand function, not measured by MRC-SS testing. It was not possible to assess physical function at awakening and testing MRC-SS at discharge was not central to the current study, which focussed on the usefulness of MRC-SS testing during the early stage of critical illness.

We observed no difference in physical function measures between groups with and without a diagnosis of ICU-AW, albeit this may be attributable to the smaller sample of patients without ICU-AW following ICU discharge. These are similar to the findings from Kleyweg*et al’s* original MRC-SS work ([7](#_ENREF_7)) who reported that in patients with higher physical function levels, the MRC-SS offered little additional information. One explanation for this change in MRC-SS without a change in functional level ([7](#_ENREF_7)), is the lack of sensitivity in the MRC grading scale to diagnose clinical weakness despite the presence of underlying electrophysiological changes ([8](#_ENREF_8)).

There are few recognised, validated outcome measures for critically ill patients on discharge from the ICU. We selected two measures that we felt represented functional activities potentially requiring rehabilitation assessment. However the weak association demonstrated could be a result of the poor validity of these measures in the post ICU population, rather than a function of the MRC-SS. Furthermore we analysed MRC-SS and physical function at differing time-points, awakening and at ICU discharge respectively, and the weak relationships observed may be influenced by improvements in performance during the interim period. Contemporaneous measurements of MRC-SS and physical function may have yielded stronger correlations, but physical function manoeuvres are often not possible at awakening and MRC-SS measurements beyond awakening limit use of the score as a predictor of both ICU and hospital outcome.

These data demonstrate that muscle strength determined using the MRC-SS does not translate into physical function ability when assessed using two common clinical measures, and has implications for the provision of rehabilitation to clinically impaired patients.

**S2. Standardised protocol for Medical Research Council-Sum score muscle strength assessment**

For the purposes of this study, a local standardised protocol was designed and agreed upon by the two clinical examiners for all MRC-SS (*Table S2a*) measurements, taking into consideration pragmatic clinical factors involved in performing assessments.Assessments were performed in either supine (*Table S2b*)or seated (*Table S2c*)depending on clinical circumstances. Screening for awakening and suitability for completing testing was completed on each occasion by each clinician.Both clinicians assessed each patient in the same position.

Despite adopting these standardised protocols, it is acknowledged that difficulty in achieving optimum patient positioning according to instructions, to adequately perform muscle testing may have limited results.This is further influenced by the ordinal nature of the MRC grading scale, and the named muscle groups included in the MRC-SS for testing.

**Table 2a:** The Medical Research Council Sum-score

| **Muscle groups** | **Medical Research Council strength scale** |
| --- | --- |
| Shoulder abduction | 0 – no visible contraction |
| Elbow flexion | 1 – visible contraction but no movement |
| Wrist extension | 2 – active movement, gravity eliminated |
| Hip flexion | 3 – active movement against gravity |
| Knee extension | 4 – active movement against resistance |
| Ankle dorsiflexion | 5 – active movement against full resistance (normal power) |

^Muscle groups measured bilaterally. At least 2 limbs are required for measurement; where certain muscle groups cannot be assessed, due to clinical or pragmatic reason, total MRC-SS are determined by extrapolating the score from the contralateral identical muscle group. Total score out of 60. ICU-AW defined as MRC-SS <48/60.^

*^Abbreviations: MRC-SS = Medical Research Council sum-score; ICU-AW = intensive care unit-acquired weakness^*

**Table S2b.**Local standardised protocol for Medical Research Council sum-score assessments on patients positioned in bed

| **Item** | **Protocol** |
| --- | --- |
| - Patient position | - Supine with 30° head elevation, with the exception of upright sitting for assessment of shoulder abduction (to allow for movement against gravity) - Remove all extra pillows - Ensure central, midline position determined by pelvic and shoulder symmetry |
| - Order of testing of limb muscle group | - Distal to proximal anti-clockwise starting with right lower limb i.e. right lower limb, left lower limb, left upper limb, right upper limb - Shoulder abduction is the last muscle group assessed following repositioning (see above) |
| - Passive range of movement assessment | - Allows for assessment of sensation, available joint range of movement, presence of velocity-dependent tonal changes that may influence measurement - Instruction to patient “Do not do anything, keep as relaxed as possible” |
| - Active performance of muscle movement - Ankle dorsiflexion - Knee extension - Hip flexion - Wrist extension - Elbow flexion - Shoulder abduction | - Allows for grading of muscle strength according to MRC scale (Table S2a) - Observe and palpate muscle group for sign of activity (Levels 0 and 1) e.g. quadriceps for knee extension - Instruction to patient “Move your (limb segment) towards (appropriate direction) as much as you can and hold it there” - Tester fixes ankle with one hand to ensure midline position - Resistance applied along dorsum of foot with opposite hand for >Grade 3 - Tester rests patient leg over their knee in flexed position, and fixes knee with one hand to ensure midline position - Resistance applied at distal two-thirds shin with opposite hand for >Grade 3 - Grade 3 = movement from supine to hip flexion achieving foot flat on bed - With hip in 90° hip flexion, resistance applied to mid thigh for >Grade 3 - Wrist is isolated by fixing the forearm above the joint line, in pronated position - Resistance applied over dorsum of hand for >Grade 3 - A pillow can be used, or the elbow can be flexed to 90° to bring the wrist joint into eyesight of patient if necessary - Tester ensures neutral midline forearm position; a pillow may be used to ensure upper arm is horizontal with body - Resistance is applied to the forearm for >Grade 3 - Elbow flexion is advised to create a shorter lever for movement, and to achieve a neutral scapular position with some horizontal flexion and external rotation as comfort requires - Grade 3 = 90° shoulder abduction - Resistance is applied to the upper arm for >Grade 3 |
| - ‘Break’ test for >Grade 3 | - Performed in mid- to inner-range of movement; this position can be demonstrated by tester - Instruction to patient “Hold your (limb segment) there, don’t let me move you’ - Force is gradually applied opposing the direction of limb segment movement, up to near maximal levels to ‘break’ patient resistance to movement - Grade 4 = On application of force, tester can ‘break’ the patient’s static position - Grade 5 = on application of force, tester unable to move the body segment without involvement of other muscle groups/change in patient body position |

**Table S2c.**Local standardised protocol for Medical Research Council sum-score assessments on patients positioned seated in a chair

| **Item** | **Protocol** |
| --- | --- |
| - Patient position | - Upright sitting, as near to 90° as possible - Remove all extra pillows - Ensure central, midline position determined by pelvic and shoulder symmetry and knee position |
| - Order of testing of limb muscle group | - Distal to proximal anti-clockwise starting with right lower limb i.e. right lower limb, left lower limb, left upper limb, right upper limb |
| - Passive range of movement assessment | - Allows for assessment of sensation, available joint range of movement, presence of velocity-dependent tonal changes that may influence measurement - Instruction to patient “Do not do anything, keep as relaxed as possible” |
| - Active performance of muscle movement - Ankle dorsiflexion - Knee extension - Hip flexion - Wrist extension - Elbow flexion - Shoulder abduction | - Allows for grading of muscle strength according to MRC scale (Table S2a) - Observe and palpate muscle group for sign of activity (Levels 0 and 1) e.g. quadriceps for knee extension - Instruction to patient “Move your (limb segment) towards (appropriate direction) - Patient sits with hips in midline, knees flexed to mid-range and heels resting on stable surface - Testers kneels by patients and fixes ankle with one hand to ensure midline position - Resistance applied along dorsum of foot with opposite hand for >Grade 3 - Patient sits with hips in midline, knees flexed to mid-range and heels resting on stable surface - Tester rests leg over their forearm to ensure support under knee in flexion, and fixes knee with one hand to ensure midline position - Resistance applied with other hand at distal two-thirds shin by applying body weight - Patient is seated with hips in passive flexion - Tester fixes at knee to ensure lower limb is in midline - Grade 3 = active movement beyond degree of passive seated hip flexion - Resistance is applied to patient’s thigh for >Grade 3 - Forearm rests in pronation on a stable surface by patient - Tester stands perpendicular to patients and isolates wrist by fixing forearm above joint line - Resistance is applied through dorsum of hand for >Grade 3 - Tested with elbow at patient’s side in neutral pronation/supination - Resistance applied to forearm for >Grade 3 - Elbow flexion is advised to create a shorter lever for movement, and to achieve a neutral scapular position with some horizontal flexion and external rotation as comfort requires - Grade 3 = 90° shoulder abduction - Tester stands wide stance perpendicular to patient - Resistance is applied to the upper arm for >Grade 3 |
| - ‘Break’ test for >Grade 3 | - Performed in mid- to inner-range of movement; this position can be demonstrated by tester - Instruction to patient “Hold your (limb segment) there, don’t let me move you’ - Force is gradually applied opposing the direction of limb segment movement, up to near maximal levels to ‘break’ patient resistance to movement - Grade 4 = On application of force, tester can ‘break’ the patient’s static position - Grade 5 = on application of force, tester unable to move the body segment without involvement of other muscle groups/change in patient body position |

**S3. Additional detail on performance and assessment of simulated Medical Research Council sum-scores**

In order to assess simulated weakness, one healthy volunteer was trained comprehensively in the Medical Research Council sum-score, including the muscle groups assessed, and the different levels of weakness evident (ranging from 0-5). After a period of practice, the healthy volunteer was then instructed to mimic the twenty reference MRC-SS, in a random order, which were then re-scored by each clinician. Clinician order of testing was randomised for the first simulated presentation, following an alternating pattern thereafter. Both clinicians adopted the standard operating protocol for performing MRC-SS, and the healthy volunteer simulated the weakness presentations in the same position as in the original.

**S4. Additional analysis of inter-observer agreement of the Medical Research Council sum-score**

Inter-observer agreementbetween clinicians for the MRC-SS in ICU patients was further determined by analysing individual muscle group scores.Intra-class correlation coefficients (ICC) were calculated using a two-way random effects for absolute agreement ([9](#_ENREF_9)) and percent agreement for individual muscle group scores. Percent agreement was calculated by dividing the total number of exact scores by the total number. Levels of agreement for each clinician for the binary outcome of weakness (MRC score <4:≥4) were determined using Cohen’s Kappa statistic using a grading system from ‘poor’ to ‘almost perfect’ agreement ([10](#_ENREF_10)). Median individual muscle group scores for each clinician were compared using Wilcoxon signed rank testing.

This process was repeated for the individual muscle group scores from the simulated presentations, and furthermore for analysing each clinician’s simulated MRC-SS results against the reference score forboth total scores and individual muscle group scores. The reference score was the original MRC-SS taken from and ICU patient. Where applicable agreement for the binary outcomes of ICU-AW (MRC-SS <48;≥48) and individual muscle group weakness (MRC score <4:≥4) were employed.Median individual muscle group scores for each clinician were compared using Wilcoxon signed rank testing.We considered a *p* value of less than 0.05 statistically significant. Analyses were performed using IBM SPSS Statistics, USA, GraphPad Prism v5 for Windows, USA and CIA for Windows, Southampton, UK.

**S5.Additional results for inter-observer agreement of the Medical Research Council sum-score in intensive care unit patients**

Inter-observer agreement of the MRC-SS in ICU patients was also determined for individual muscle groups tested (*Table S5a).*  Maximum percent agreement between clinicians was 75% for right knee extension and left ankle dorsiflexion. The strongest ICC for MRC scores of individual muscle groups was for left ankle dorsiflexion (0.86, 95% CI 0.68-0.94). Agreement for identification of weakness (MRC score <4:≥4) reported using Kappa statistic, ranged from 0.43-0.88. There was no evident trend for proximal and distal muscle groups.

**Table S5a.** Inter-observer agreement for individual muscle group scores for intensive care unit patients

| **Muscle group** | **Kappa (95% CI)** | **ICC (95% CI)** | **% agreement** |
| --- | --- | --- | --- |
| **Shoulder abduction L** | 0.8 (0.54 – 1.06) | 0.85 (0.67 – 0.94) | 70 |
| **Shoulder abduction R** | 0.68 (0.35 – 1.01) | 0.74 (0.46 – 0.89) | 60 |
| **Elbow flexion L** | 0.69 (0.29 – 1.1) | 0.69 (0.36 – 0.86) | 65 |
| **Elbow flexion R** | 0.88 (0.64 – 1.11) | 0.73 (0.43 – 0.88) | 65 |
| **Wrist extension L** | 0.74 (0.39 – 1.08) | 0.74 (0.46 – 0.89) | 60 |
| **Wrist extension R** | 0.47 (0.06 – 0.87) | 0.64 (0.27 – 0.84) | 45 |
| **Hip flexion L** | 0.56 (0.18 – 0.95) | 0.6 (0.22 – 0.82) | 55 |
| **Hip flexion R** | 0.43 (0.001 -0.87) | 0.76 (0.48 – 0.9) | 60 |
| **Knee extension L** | 0.47 (0.06 – 0.87) | 0.63 (0.28 – 0.84) | 35 |
| **Knee extension R** | 0.57 (0.18 – 0.95) | 0.69 (0.37 – 0.86) | 75 |
| **Ankle dorsiflexion L** | 0.83 (0.5 – 1.16) | 0.86 (0.68 – 0.94) | 75 |
| **Ankle dorsiflexion R** | 0.86 (0.58 – 1.13) | 0.77 (0.52 – 0.9) | 45 |

^Kappa statistic using binary outcome of clinical weakness (Medical Research Council score <4; ≥4). n=20^

*^Abbreviations: L = left. R = right. CI = confidence interval. ICC = intra-class correlation coefficient^*

Minimum, maximum and median scores for each clinician’s individual muscle group score for ICU patients are shown in Table S5b. No significant difference between each clinicians’ median score for any muscle group was evident with the exception of right wrist extension (p=0.03).

**Table S5b.** Individual muscle group scores for intensive care unit patients

| **Muscle group** | **Rater 1** | | | **Rater 2** | | | **p value** |
| --- | --- | --- | --- | --- | --- | --- | --- |
|  | **Min** | **Max** | **Median (IQR)** | **Min** | **Max** | **Median (IQR)** |  |
| **Shoulder abduction L** | 1 | 5 | 4 (2-4) | 2 | 5 | 3.5 (3-4) | 0.48 |
| **Shoulder abduction R** | 1 | 5 | 4 (2-4) | 2 | 5 | 4 (3-4) | 0.15 |
| **Elbow flexion L** | 2 | 5 | 4 (4-4) | 2 | 4 | 4 (3.25-4) | 0.07 |
| **Elbow flexion R** | 2 | 5 | 4 (3.25-4) | 2 | 5 | 4 (3-5) | 0.59 |
| **Wrist extension L** | 2 | 5 | 4 (4-4) | 1 | 5 | 4 (3-4) | 0.15 |
| **Wrist extension R** | 2 | 5 | 4 (2.25-4) | 2 | 5 | 4 (4-5) | 0.03 |
| **Hip flexion L** | 2 | 5 | 4 (2.25-5) | 2 | 5 | 4 (3-4) | 1.0 |
| **Hip flexion R** | 2 | 5 | 4 (2-5) | 2 | 5 | 4 (3-4) | 0.82 |
| **Knee extension L** | 1 | 5 | 4 (2-5) | 2 | 5 | 4 (3-4.75) | 0.42 |
| **Knee extension R** | 2 | 5 | 4 (2-5) | 2 | 5 | 4 (2-5) | 0.59 |
| **Ankle dorsiflexion L** | 1 | 5 | 4 (4-5) | 0 | 5 | 4 (4-5) | 1.0 |
| **Ankle dorsiflexion R** | 1 | 5 | 4 (4-5) | 2 | 5 | 4 (2.5-4.75) | 0.18 |

^p values derived from Wilcoxon signed rank test for equality of median score. n=20.^

*^Definitions: Min = minimum score. Max =maximum score. IQR = interquartile range.^*

**S6.Additional results for inter-observer agreement of the Medical Research Council sum-score in simulated presentations**

Inter-observer agreement between the two clinicians for individual muscle groups assessed in simulated MRC-SS presentations is shown in *Table S6a*. A minimum of 85% agreement between clinicians was demonstrated and ICC ranged from 0.93 to 1.0. For the majority of muscle groups, perfect agreement was demonstrated for the diagnosis of clinical weakness (MRC score <4; ≥4) as indicated by Kappa statistic of 1.0. The weakest level of agreement was for right hip flexion (Kappa 0.74).

**Table S6a.** Inter-observer agreement for individual muscle group sores for simulated presentations

| **Muscle group** | **Kappa (95% CI)** | **ICC (95% CI)** | **% agreement** |
| --- | --- | --- | --- |
| **Shoulder abduction L** | 1.0 (1.0 – 1.0) | 1.0 (1.0-1.0) | 100 |
| **Shoulder abduction R** | 1.0 (1.0 – 1.0) | 0.98 (0.95 – 0.99) | 95 |
| **Elbow flexion L** | 0.86 (0.58 – 1.13) | 0.96 (0.91 – 0.99) | 95 |
| **Elbow flexion R** | 1.0 (1.0 – 1.0) | 0.98 (0.94 – 0.99) | 95 |
| **Wrist extension L** | 0.88 (0.64 – 1.11) | 0.95 (0.88 – 0.98) | 90 |
| **Wrist extension R** | 1.0 (1.0 – 1.0) | 0.98 (0.95 – 0.99) | 95 |
| **Hip flexion L** | 1.0 (1.0 – 1.0) | 1.0 (1.0-1.0) | 100 |
| **Hip flexion R** | 0.74 (0.39 – 1.08) | 0.93 (0.82 – 0.97) | 85 |
| **Knee extension L** | 1.0 (1.0 – 1.0) | 0.97 (0.91 – 0.99) | 90 |
| **Knee extension R** | 1.0 (1.0 – 1.0) | 0.97 (0.92 – 0.99) | 90 |
| **Ankle dorsiflexion L** | 0.86 (0.58 – 1.13) | 0.97 (0.93 – 0.99) | 90 |
| **Ankle dorsiflexion R** | 1.0 (1.0 – 1.0) | 0.97 (0.94 – 0.99) | 90 |

^Kappa statistic using binary outcome of clinical weakness (Medical Research Council score <4; ≥4). n=20^

*^Abbreviations: L = left. R = right. CI = confidence interval. ICC = intra-class correlation coefficient.^*

Minimum, maximum and median scores for each clinician’s individual muscle group score on simulated presentations are shown in *Table S6b*. No significant difference between both clinicians’ median scores for individual muscle groups was evident, and for two muscle groups (left shoulder abduction and left hip flexion) both sets of scores were identical.

**Table S6b*.*** Individual muscle group scores for simulated presentations

| **Muscle group** | **Rater 1** | | | **Rater 2** | | | **p value** |
| --- | --- | --- | --- | --- | --- | --- | --- |
|  | **Min** | **Max** | **Median (IQR)** | **Min** | **Max** | **Median (IQR)** |  |
| **Shoulder abduction L** | 1 | 5 | 4 (2-4) | 1 | 5 | 4 (2-4) | --- |
| **Shoulder abduction R** | 1 | 5 | 4 (2.25-4) | 1 | 5 | 4 (2.25-4) | 1.0 |
| **Elbow flexion L** | 2 | 5 | 4 (3.25-4) | 2 | 5 | 4 (4-4) | 1.0 |
| **Elbow flexion R** | 2 | 5 | 4 (3-5) | 2 | 5 | 4 (3-5) | 1.0 |
| **Wrist extension L** | 1 | 5 | 4 (3-4) | 1 | 5 | 4 (3.25-4) | 0.35 |
| **Wrist extension R** | 2 | 5 | 4 (3-4.75) | 2 | 5 | 4 (3-5) | 1.0 |
| **Hip flexion L** | 2 | 5 | 4 (3-4) | 2 | 5 | 4 (3-4) | --- |
| **Hip flexion R** | 2 | 5 | 4 (4-5) | 2 | 5 | 4 (3-4.75) | 0.15 |
| **Knee extension L** | 2 | 5 | 4 (2-4) | 2 | 5 | 4 (2-5) | 0.35 |
| **Knee extension R** | 2 | 5 | 4 (2-5) | 2 | 5 | 4 (2-5) | 0.35 |
| **Ankle dorsiflexion L** | 1 | 5 | 4 (2.25-4) | 1 | 5 | 4 (2.25-4) | 1.0 |
| **Ankle dorsiflexion R** | 1 | 5 | 4.5 (2.5-5) | 1 | 5 | 4 (2.5-5) | 1.0 |

^p values derived from Wilcoxon signed rank test for equality of median score. --- = both clinicians’ median scores identical therefore unable to compute result. n=20.^

*^Definitions: Min = minimum score. Max =maximum score. IQR = interquartile range.^*

**S7. Additional results for inter-observer agreement between each clinician and the reference Medical Research Council sum-score**

Inter-observer agreement of each clinician’s MRC-SS from simulated presentations was compared against the reference score. Both clinicians demonstrated an ICC of 1.0 (95% CI 1.0-1.0), percent agreements of 65 and 70% and perfect agreement for diagnosis of ICU-AW with Kappa statistics of 1.0 (95% CI 1.0-1.0).*Tables S7a* and *S7b* reports each clinician’s individual muscle group scores against the reference MRC score. High levels of agreement were evident for both clinicians.

**Table S7a.** Inter-observer agreement between Clinician 1 and reference scores for individual muscle groups

| **Muscle group** | **Kappa (95% CI)** | **ICC (95% CI)** | **% agreement** |
| --- | --- | --- | --- |
| **Shoulder abduction L** | 0.9 (0.71 – 1.09) | 0.98 (0.95 – 0.99) | 95 |
| **Shoulder abduction R** | 0.9 (0.7 – 1.09) | 0.98 (0.95 – 0.99) | 95 |
| **Elbow flexion L** | 0.86 (0.58 – 1.13) | 0.96 (0.91 – 0.99) | 95 |
| **Elbow flexion R** | 1.0 (1.0 – 1.0) | 1.0 (1.0 – 1.0) | 100 |
| **Wrist extension L** | 0.88 (0.64 – 1.11) | 0.98 (0.94 – 0.99) | 95 |
| **Wrist extension R** | 1.0 (1.0 – 1.0) | 1.0 (1.0 – 1.0) | 100 |
| **Hip flexion L** | 1.0 (1.0 – 1.0) | 1.0 (1.0 – 1.0) | 100 |
| **Hip flexion R** | 0.86 (0.58 – 1.13) | 0.95 (0.88 – 0.98) | 90 |
| **Knee extension L** | 1.0 (1.0 – 1.0) | 0.97 (0.91 – 0.99) | 90 |
| **Knee extension R** | 1.0 (1.0 – 1.0) | 1.0 (1.0 – 1.0) | 100 |
| **Ankle dorsiflexion L** | 1.0 (1.0 – 1.0) | 0.99 (0.97 – 0.99) | 95 |
| **Ankle dorsiflexion R** | 1.0 (1.0 – 1.0) | 0.97 (0.94 – 0.99) | 90 |

Kappa statistic using binary outcome of clinical weakness (Medical Research Council score <4;≥4).

*Definitions: L = left. R = right. CI = confidence interval. ICC = intra-class correlation coefficient*

**Table S7b.** Inter-observer agreement between Clinician 2 and reference scores for individual muscle groups

| **Muscle group** | **Kappa (95% CI)** | **ICC (95% CI)** | **% agreement** |
| --- | --- | --- | --- |
| **Shoulder abduction L** | 0.9 (0.71 – 1.09) | 0.98 (0.95 – 0.99) | 95 |
| **Shoulder abduction R** | 0.9 (0.7 – 1.09) | 0.96 (0.89 – 0.98) | 90 |
| **Elbow flexion L** | 1.0 (1.0 – 1.0) | 1.0 (1.0-1.0) | 100 |
| **Elbow flexion R** | 1.0 (1.0 – 1.0) | 0.98 (0.94 – 0.99) | 95 |
| **Wrist extension L** | 1.0 (1.0 – 1.0) | 0.98 (0.94 – 0.99) | 95 |
| **Wrist extension R** | 1.0 (1.0 – 1.0) | 0.98 (0.95 – 0.99) | 95 |
| **Hip flexion L** | 1.0 (1.0 – 1.0) | 1.0 (1.0-1.0) | 100 |
| **Hip flexion R** | 0.88 (0.64 – 1.11) | 0.98 (0.94 – 0.99) | 90 |
| **Knee extension L** | 1.0 (1.0 – 1.0) | 1.0 (1.0-1.0) | 100 |
| **Knee extension R** | 1.0 (1.0 – 1.0) | 0.97 (0.92 – 0.99) | 90 |
| **Ankle dorsiflexion L** | 0.86 (0.58 – 1.13) | 0.99 (0.97 – 1.0) | 95 |
| **Ankle dorsiflexion R** | 1.0 (1.0 – 1.0) | 1.0 (1.0-1.0) | 100 |

Kappa statistic using binary outcome of clinical weakness (Medical Research Council score <4;≥4).

*Definitions: L = left. R = right. CI = confidence interval. ICC = intra-class correlation coefficient*

**S8. Additional results for Receiver-Operator Curve analysis of Medical Research Council sum-score**

We performed receiver-operator curve (ROC) analysis on the 65 awakening MRC-SS measurements for each clinical outcome (ICU and hospital mortality and ICU and hospital length of stay) to assess the sensitivity and specificity at levels of MRC-SS from zero to 60. Data from these analyses are reported in *Table S9*.

**Table S8.**Receiver-operator curve analyses of Medical Research Council sum-score at awakening and clinical outcome

| **Clinical outcome** | **MRC-SS (/60)** | **Sensitivity (%)** | **Specificity (%)** | **AUC (95%CI)** | **p** |
| --- | --- | --- | --- | --- | --- |
| **ICU mortality** | <29.5 | 62.5 | 70.2 | 0.63 (0.42-0.83) | 0.26 |
| **ICU LOS** | <35 | 64.3 | 64.9 | 0.69 (0.56-0.82) | 0.009 |
| **Hospital mortality** | <35 | 62.5 | 57.1 | 0.55 (0.4-0.7) | 0.55 |
| **Hospital LOS** | <36.5 | 60.5 | 63.0 | 0.65 (0.51-0.79) | 0.04 |

MRC-SS at awakening, n=65. Cut-offs reported represent those at which greatest levels of sensitivity and specificity were identified.

*Definitions: MRC-SS = Medical Research Council sum score. ICU = Intensive Care Unit. LOS = length of stay. AUC = area under the curve*.

**References**

1. Ali NA, O'Brien JM, Jr., Hoffmann SP, Phillips G, Garland A, Finley JCW, Almoosa K, Hejal R, Wolf KM, Lemeshow S, Connors AF, Jr., Marsh CB, for The Midwest Critical Care Consortium. Acquired weakness, handgrip strength, and mortality in critically ill patients. *Am J Resp Crit Care Med* 2008;178:261-268.

2. Hermans G, Clerckx B, Vanhullebusch T, Segers J, Vanpee G, Robbeets C, Casaer MP, Wouters P, Gosselink R, Van Den Berghe G. Interobserver agreement of medical research council sum-score and handgrip strength in the intensive care unit. *Muscle a d Nerve* 2012;45:18-25.

3. Mahoney F, Barthel D. Functional evaluation: The barthel index. *Md State Med J* 1965;14:56-61.

4. Smith R. Validation and reliability of the elderly mobility scale. *Physiotherapy* 1994;80:744-747.

5. Stevens RD, Marshall SA, Cornblath DR, Hoke A, Needham DM, de Jonghe B, Ali NA, Sharshar T. A framework for diagnosing and classifying intensive care unit-acquired weakness. *Crit Care Med* 2009;37:S299-308.

6. Clinical assessment recommendations. The American Society of Hand Therapists; 1992.

7. Kleyweg RP, van der Meche FG, Schmitz PI. Interobserver agreement in the assessment of muscle strength and functional abilities in guillain-barre syndrome. *Muscle Nerve* 1991;14:1103-1109.

8. Mills KR. Wasting, weakness and the mrc scale in the first dorsal interosseous miscle. *Journal of Neurology Neurosurgery and Psychiatry* 1997;62:541-542.

9. Shrout PE, Fleiss JL. Intraclass correlations: Uses in assessing rater reliability. *Psychol Bull* 1979;86:420-428.

10. Guggenmoos-Holzmann I. The meaning of kappa: Probabilistic concepts of reliability and validity revisited. *J Clin Epidemiol* 1996;49:775-782.
